# Antioxidant Activities of an Exopolysaccharide (DeinoPol) Produced by the Extreme Radiation-Resistant Bacterium *Deinococcus radiodurans*

**DOI:** 10.1038/s41598-019-56141-3

**Published:** 2020-01-09

**Authors:** Shun Mei Lin, Chan Yu Baek, Jong-Hyun Jung, Woo Sik Kim, Ha-Yeon Song, Ji Hee Lee, Hyun Jung Ji, Yong Zhi, Bo Sun Kang, Yong-Sun Bahn, Ho Seong Seo, Sangyong Lim

**Affiliations:** 1 Research Division for Radiation Science, Korea Atomic Energy Research Institute, Jeongeup, Republic of Korea; 2 R&D Center, MediNResearch, Jeongeup, Republic of Korea; 3 Department of Biotechnology, College of Life Science and Biotechnology, Yonsei University, Seoul, Republic of Korea; 4 Department of Radiation Science, University of Science and Technology, Daejeon, Republic of Korea; 5 Department of Radiological Science, Konyang University, Daejeon, Republic of Korea

**Keywords:** Biopolymers, Microbial ecology, Molecular medicine

## Abstract

*Deinococcus radiodurans* shows extreme resistance to a range of remarkable environmental stresses. Deinococcal exopolysaccharide (DeinoPol) is a component of the cell wall, but its role in stress resistance has not yet been well-described. In this study, we isolated and characterized DeinoPol from *Deinococcus radiodurans* R1 strain and investigated its application as an antioxidant agent. Bioinformatic analysis indicated that *dra0033*, encoding an ExoP-like protein, was involved in DeinoPol biosynthesis, and *dra0033* mutation significantly decreased survival rates in response to stresses. Purified DeinoPol consists of different monosaccharides and has a molecular weight of approximately 80 to 100 kDa. DeinoPol also demonstrates highly protective effects on human keratinocytes in response to stress-induced apoptosis by effectively scavenging ROS. Taken together, these findings indicate that DeinoPol is the first reported deinococcal exopolysaccharide that might be used in cosmetics and pharmaceuticals as a safe and attractive radical scavenger.

## Introduction

Exposure to ultraviolet (UV) radiation can generate reactive oxygen species (ROS), resulting in DNA, lipid, and protein damage in cells and tissues such as the skin^1^, which becomes mildly inflamed, leading to photo-aging and carcinogenesis^2,3^. The ROS and free radicals that are generated tend to stabilize themselves by scavenging electrons from biomolecules, triggering the activation of many cellular signaling cascades, including apoptosis and necrosis^4^. A broad spectrum of natural antioxidants such as flavonoids, polyphenols, and sterols may be deployed to lessen these effects^5,6,7^. Given their low negative effect on organisms and the environment, natural products with photo-protective properties are increasingly used to prevent radiation-induced skin damage.

Recent research has shown that polysaccharides from natural products possess wide-ranging beneficial therapeutic effects and health-promoting properties^8^. Bacterial exopolysaccharides (EPSs) are mostly nontoxic natural biopolymers with extensive applications in areas such as pharmaceuticals, nutraceuticals and functional foods, cosmetics, and insecticides^9–13^. An extreme resistance to ionizing radiation, desiccation, UV radiation, oxidizing agents, and electrophilic mutagens has been observed in *Deinococcus radiodurans*^14^. This is attributed to an enhancement of functional redundancies in both efficient protection from ROS by protein and lipid modifications and in DNA repair mechanisms^15^. These characteristics are of interest in developing it for bioremediation of radioactive wastes and biomolecule production. The deinococcal cell envelope consists of a fragile, soft layer containing carotenoids, lipids, proteins, and EPSs^16,17^. However, whether it has protective capacities against ROS-induced cellular damage remains poorly understood.

The present study aimed to elucidate the role of the deinococcal EPS, DeinoPol in oxidative stress, as well as to characterize its sugar composition and antioxidant effects, including scavenging effects on hydroxyl radicals and anti-apoptosis activities in human keratinocytes.

## Results

### Identification and characterization of DeinoPol synthesis genes in *D. radiodurans*

**Figure 1:**
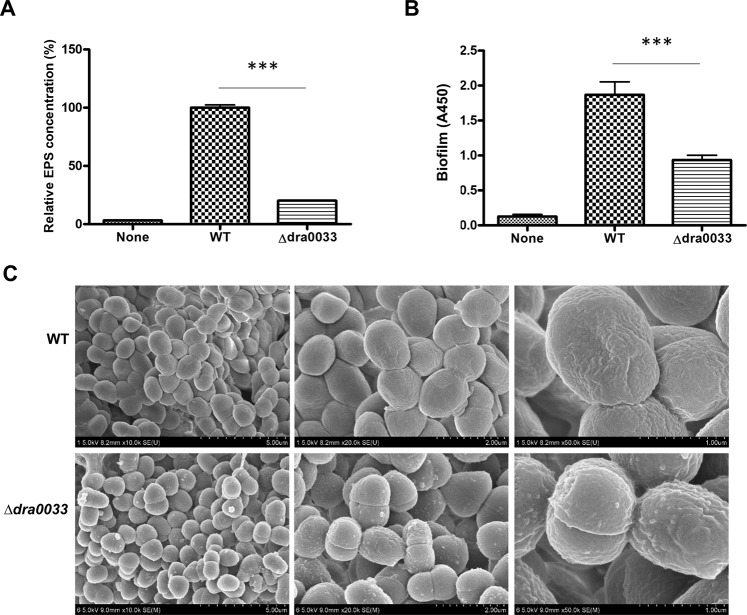
Characterization of DeinoPol production in *D. radiodurans* R1 and *dra0033* mutant. (**A)** Relative quantification of DeinoPol production in *D. radiodurans* R1 and its isogenic mutant (Δ*dra0033*). *Deinococcus* was cultured in TY broth for 48 h at 30 °C, and DeinoPol was precipitated with 80% ethanol at 4 °C. DeinoPol was hydrolyzed and reacted with anthrone and absorbance was read at 490 nm. (**B)** Relative biofilm formation of *D. radiodurans* R1 and its isogenic mutant (Δ*dra0033*). Bacteria were seeded in a 96-well plate and incubated for 48 h at 30 °C. Biofilm formation was measured by staining with 1% crystal violet, and absorbance was read at 450 nm. (**C)** Surface layers of *D. radiodurans* R1 and its isogenic mutant (Δ*dra0033*). Surface-expressed DeinoPol was visualized by scanning electron microscopy (SEM). The rough surface and septa structure indicated a decreased amount of EPS on the deinococcal surface.

In previous studies, an ExoP-like protein was shown as a critical regulator of EPS synthesis, directly or indirectly. PSI-BLAST analysis of the predicted amino acid sequence of the ExoP-like protein of *Pseudomonas solanacearum* indicated that DRA0033 exhibited 95/329 (29%) amino acid identity^18^. To examine whether *dra0033* was involved in EPS synthesis, we first constructed a *dra0033*-deficient strain in *D. radiodurans* reference strain R1 and determined the amount of DeinoPol in the culture supernatants using an ethanol-precipitation method. As shown in Fig. 1A, the mutant produced 79.8% less DeinoPol than that by the WT strain. Bacterial EPSs enable the attachment and subsequent formation of biofilm of bacteria on surfaces^9^. When WT and ∆*dra0033* strains were cultured on polystyrene plates for 3 days, the amount of biofilm formation by ∆*dra0033* was significantly lower compared to that by WT, suggesting that inefficient DeinoPol production through deleting the *dra0033* gene led to reduced attachment and propagation of *D. radiodurans* on surfaces and, therefore, reduced biofilm formation (Fig. 1B). We also compared the extracellular structures using scanning electron microscopy (SEM). The WT strain showed a smooth surface and no clear septa structure, indicating that the bacteria were likely surrounded by a layer of EPS, whereas ∆*dra0033* displayed a rough surface, clear septa, and small rippled-grape skin (Fig. 1C). All these data suggested that *Deinococcus* produces EPS and DRA0033 is likely to be directly or indirectly involved in DeinoPol synthesis.

### Involvement of DeinoPol in stress resistance of *D. radiodurans*

**Figure 2:**
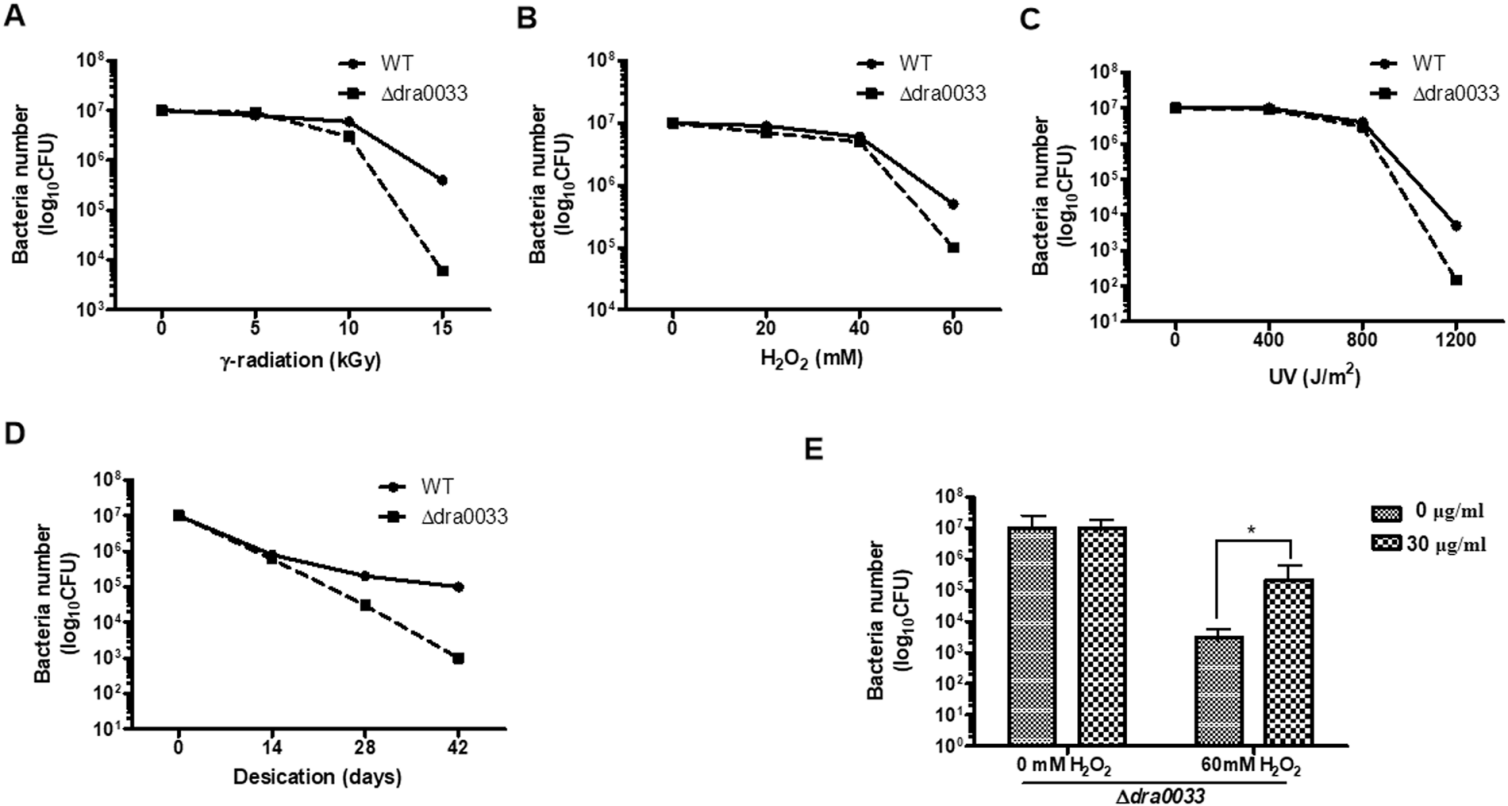
Effect of DeinoPol expression on the resistance of *D. radiodurans* R1. (**A–D)** Survival curves for *D. radiodurans* R1 and Δ*dra0033*. Mid-log phase of *D. radiodurans* R1 and its isogenic mutant (Δ*dra0033*) were exposed to γ-radiation (**A**), hydrogen peroxide for 1 h (**B**), UVC (**C**) or desiccation (**D**). Surviving bacteria were calculated by plating on TGY agar plates followed by serial dilution. (**E)** Enhancement of the survival of Δ*dra0033* against hydrogen peroxide stress with addition of exogenous purified DeinoPol (0 or 30 μg/mL). Mid-log phase Δ*dra0033* was pretreated with DeinoPol (30 μg/mL) for 30 min followed by treatment with 60 mM hydrogen peroxide for 1 h. Surviving bacteria were calculated by plating on TGY agar plates followed by serial dilution. Data are mean ± standard deviation. Asterisks indicate significant difference between Δ*dra0033* pre-treated with 0 and 30 μg/mL DeinoPol. *P < 0.05.

Since EPSs are part of a strategy for surviving adverse stress conditions^19,20^, we compared the survival ability of the WT and ∆*dra0033* variants in response to external stresses. As shown in Fig. 2A–D, WT strain was extremely resistant to γ-radiation, H_2_O_2_, UV exposure, and desiccation, whereas a reduction of survival ability was observed in the ∆*dra0033* variant.

To directly investigate the effect of DeinoPol in *D. radiodurans* R1, ∆*dra0033* was pre-treated with purified DeinoPol (30 μg/mL) for 30 min followed by incubation with a high concentration of H_2_O_2_ (60 mM) for 60 min. The untreated ∆*dra0033* strain were reduced in number by approximately 3.62 log after H_2_O_2_ treatment, whereas only 1.77 log reduction was observed in DeinoPol pre-treated ∆*dra003*3 (Fig. 2E). These data indicate that DeinoPol production might be required for deinococcal resistance to extreme abiotic stresses.

### Biochemical analysis of DeinoPol

**Figure 3:**
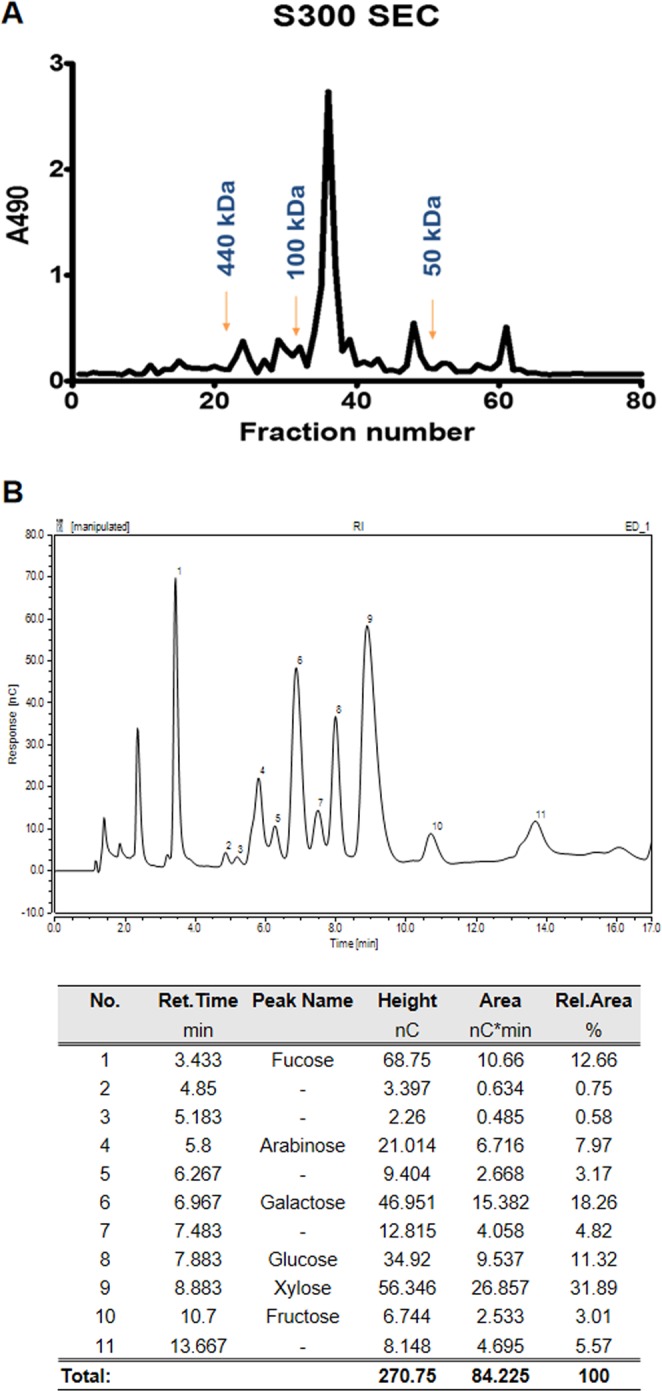
Biochemical characterization of DeinoPol. **(A)** Gel filtration chromatography of DeinoPol. Purified DeinoPol (10 mg) was fractionated on Sephacryl S-300HR. The x-axis indicates 5 mL of each fraction. The column was equilibrated with Tris buffer with 0.1 M NaCl at a flow rate of 0.5 mL/min. The y-axis indicates the peaks of eluted DeinoPol as measured by anthrone reaction with absorbance read at 490 nm. (**B)** Sugar profile of DeinoPol by Bio-LC. Ten mg dried DeinoPol was hydrolyzed and analyzed on a CarboPac TM PA1 column with a HPAEC-PAD system. The x- and y-axis indicate the retention time and integrated response ratio, respectively.

To determine the biochemical characteristics of DeinoPol, ethanol precipitation and size-exclusion chromatography were performed on deinococcal culture supernatant, followed by measurement of the eluted polysaccharide with anthrone reaction analysis^21^. As shown in Fig. 3A, DeinoPol eluted as a 80–100 kDa molecule forming a single, symmetrical peak, indicating that DeinoPol is likely to be homogenous and has relatively lower molecular weight than those of other well-known bacterial EPSs. Eluted DeinoPol contained 89.9% polysaccharide, 8.8% protein, and 1.3% DNA. Protein and DNA concentrations were calculated by measuring the optical density at 280 nm and 260 nm, respectively.

The major oligosaccharide composition of DeinoPol was analyzed using BIO-LC mass spectrometry^19^. We found six major monosaccharides: xylose, galactose, fucose, glucose, arabinose, and fructose, with molar ratios of 10.6:6.1:4.2:3.8:2.6:1.0 (Fig. 2B). In addition, five unknown sugar peaks were detected, accounting for 14.89% of the total carbohydrates. No identical or similar oligosaccharide composition of bacterial polysaccharide structure was found in the Bacterial Carbohydrate Structure DataBase (csdb.glcoscience.ru), indicating that this is the first report of DeinoPol as a novel EPS.

### Protective effect of DeinoPol against gamma-irradiation

**Figure 4:**
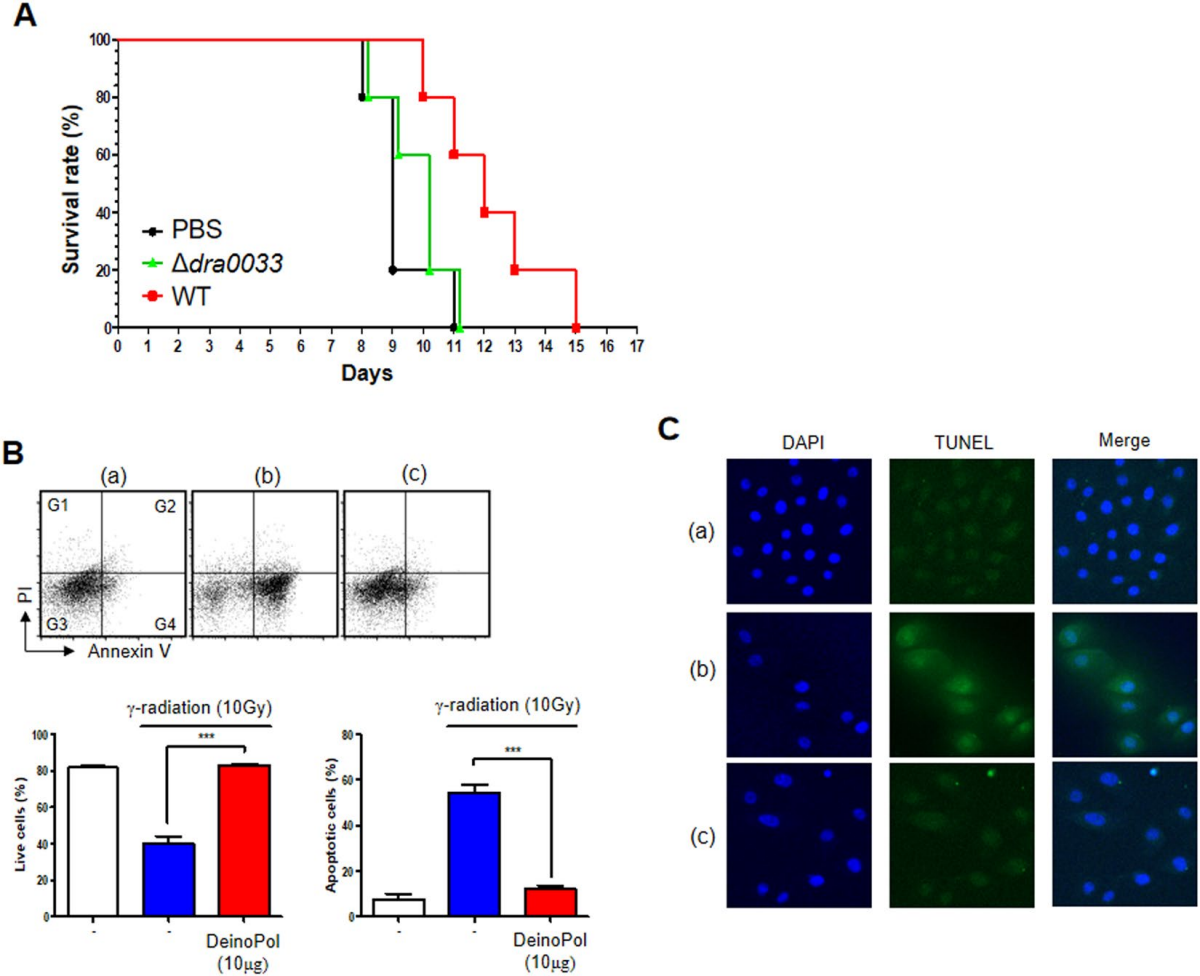
Effect of DeinoPol on γ-radiation-induced cell death. (**A)** Mice protected by injection of DeinoPol-expressing WT strain. Mice (n = 10) were injected with 10^8^ CFU of WT or Δ*dra0033* strain followed by gamma-irradiation (10 Gy). Mouse survival was monitored for 17 days. (**B)** Quantitative analysis of γ-radiation-induced apoptosis was performed by flow cytometry using NHEK-Ad cells. Cells were treated with medium alone (a), medium with 10 Gy γ-radiation (b), or 10 μg DeinoPol with 10 Gy γ-radiation (c) followed by incubating for 2 h. Cells were then subjected to flow cytometry after Annexin V-FITC/PI staining. Representative Annexin V and PI dot plots of 10,000 total cells. Quadrant 1 (Q1) contained necrotic cells (Annexin V negative and PI positive), Q2 represented the late stages of apoptosis (Annexin V and PI positive), Q3 contained living cells without signs of apoptosis (Annexin V and PI negative), and Q4 showed early stages of apoptosis (Annexin V positive and PI negative). Corresponding living (Q3) and apoptotic cell (Q2 + Q4) populations for each treatment group were analyzed. Data are presented as mean ± SD of three independent experiments. *P < 0.05. (**C**) Effect of DeinoPol on γ-radiation-induced DNA fragmentation in apoptotic cells. Cells were irradiated with 10 Gy followed by incubation for 2 h. The TUNEL assay was carried out to assess apoptotic cells after the treatments. Green fluorescent staining indicates positive apoptotic cells, and DAPI (blue) staining was used as a nuclear stain.

EPSs purified from *Bacillus* sp. isolated from the desert has high antioxidant activity *in vitro*^20^. We, therefore, investigated whether *D. radiodurans* affected the survival of γ-irradiated mice. Mice (n = 5) were injected with live WT or ∆*dra0033* (10^8^ CFU/mice) intraperitoneally (i.p.) and then subjected to γ-radiation at 10 Gy. Notably, live *D. radiodurans* showed no severe invasiveness or organ colonization (spleen, liver, kidney) in mice upon inoculation with 10^8^ CFU (Supplementary Fig. 1). All mice inoculated with either PBS or ∆*dra0033* died at 11 days after irradiation (Fig. 4A), whereas those inoculated with WT strain showed a significantly delayed death, indicating that DeinoPol is likely to provide a protective effect against lethal doses of irradiation.

To evaluate the effect of DeinoPol in radiation-induced cell death, human primary adult keratinocytes (NHEK-Ad) were irradiated at 10 Gy in the presence of DeinoPol (10 μg), and the apoptosis ratio was measured by staining with propidium iodide (PI) and Annexin V (Fig. 4B). When cells were irradiated (panel b), most were located in gates G2 (5.1%) and G4 (46.0%), indicating that radiation effectively induced early and late apoptosis. However, irradiated cells pretreated with DeinoPol (panel c) showed significant reduction in the ratio of apoptotic cells (G2 and G4) and increase in the ratio of live cells (77.6%; G3), which was similar to that of non-irradiated cells [(panel a): 80.2%], indicating that DeinoPol has the potential to protect cells from death induced by irradiation. Moreover, TUNEL assay was used to evaluate the level of apoptotic DNA fragmentation by radiation induced ROS (Fig. 4C). When cells were irradiated (panel b), fragmented DNA (bright green) was detected inside the cells. In contrast, irradiated cells pretreated with DeinoPol (panel c) showed the reduced green signal which was comparable level with non-irradiated cells (panel a).

### Reduction of ROS-induced cell death by treatment with DeinoPol

**Figure 5:**
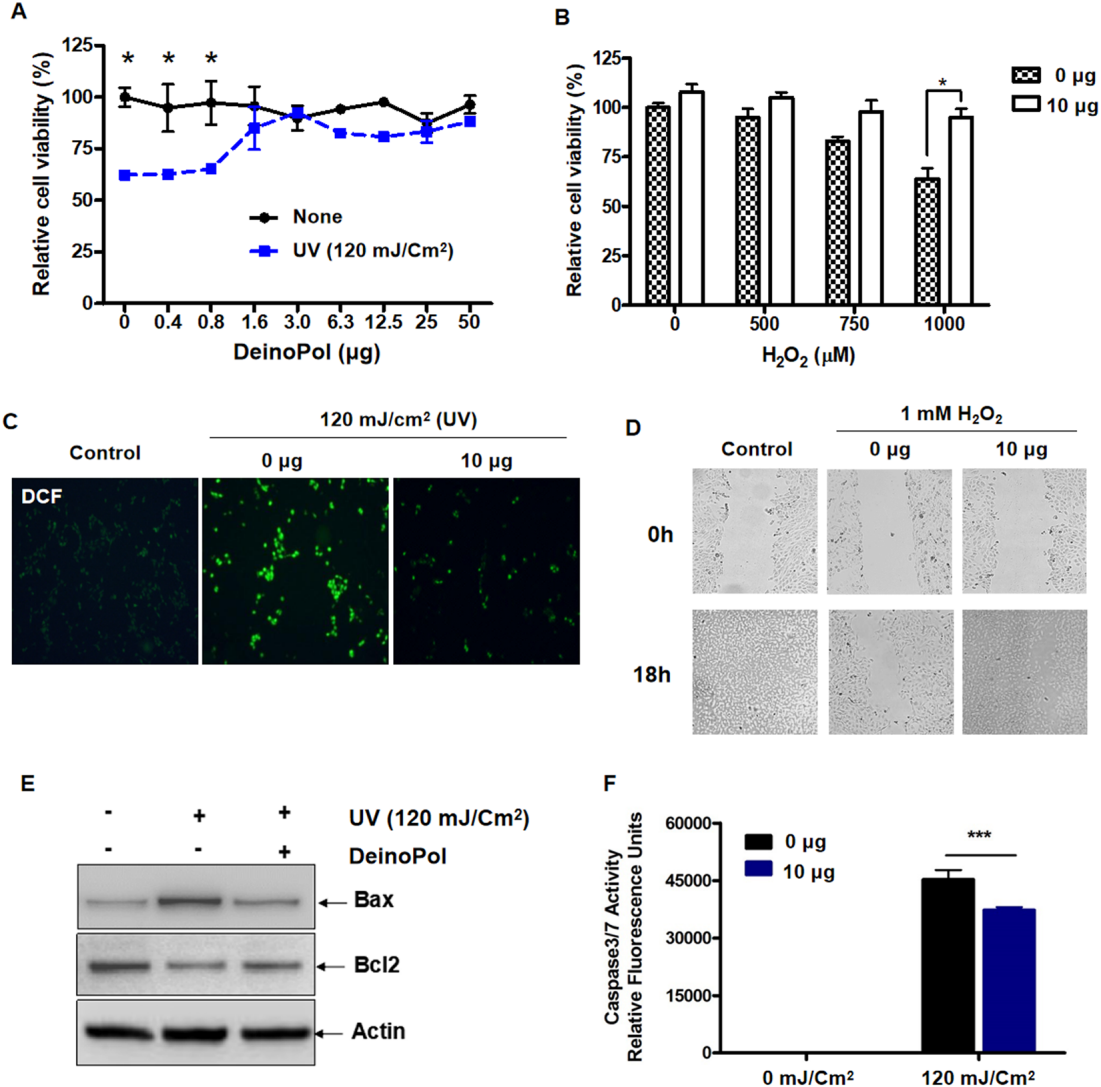
Enhancement of keratinocyte viability and proliferation by DeinoPol. (**A,B)** Effect of DeinoPol on viability of NHEK-Ad cells. A NHEK-Ad monolayer was pre-treated with the indicated concentration of DeinoPol followed by irradiation with UVB (**A**) or treatment with hydrogen peroxide (**B**). **(C)** ROS-scavenging effect of DeinoPol. NHEK-Ad cells were irradiated with 120 mJ/cm^2^ UVB in the presence of 10 μg DeinoPol followed by incubation with 5 μM H2DCFDA for 2 h. Fluorescence intensity was visualized by fluorescence microscope. (**D)** Effect of DeinoPol on NHEK-Ad proliferation by scratch wound healing assay. Scratched HaCaT cells were treated with 10 μg DeinoPol and 1 mM hydrogen peroxide. After 18 h incubation, photos of the scratched monolayer were captured. (**E)** Bax and Bcl-2 expression of UVB (120 mJ/cm^2^)-irradiated NHEK-Ad were analyzed in the presence of DeinoPol (10 μg) by western blotting. (**F)** DeinoPol reduced Caspase-3/7 activity induced by UVB. NHEK-Ad cells were treated with DeinoPol (10 μg) followed by irradiation with UVB (120 mJ/cm^2^). Caspase 3/7 activity was determined by measuring the enzyme kinetics at 2 h. Data are mean ± standard deviation. *P < 0.05, **P < 0.005, ***P < 0.001 compared with DeinoPol untreated group.

Next, since ROS such as peroxides, superoxide (^•^O^−^_2_), hydroxyl radical (^•^OH), singlet oxygen, are significantly increased owing to environmental stress, such as drought, salinity, chilling, nutrient deficiency, metal toxicity and UV radiation^22–24^, we further investigated the role of DeinoPol in minimizing cell damage induced by UV radiation and H_2_O_2_. NHEK-Ad cells were pre-treated with DeinoPol, followed by irradiation with UVB (120 mJ/cm^2^). At 12 h after irradiation, cell viability was analyzed by CCK-8 assay. In contrast to non-treated cells, pre-treated cells presented a steadily increasing viability until saturation was observed at approximately 3.0 μg/mL, similar to that of non-irradiated cells (Fig. 5A). When NHEK-Ad cells were directly sensitized with exogenous H_2_O_2_, cell viability decreased in a dose-dependent manner (Fig. 5B). At 1000 μM H_2_O_2_, cell viability was 41.3% lower than that in untreated cells, but no significant reductions in cell viability were found in DeinoPol (10 µg) pre-treated cells. Notably, no cytotoxicity was found in NHEK-Ad cells treated with over 1000 μg/mL of DeinoPol (Supplementary Fig. 2).

Intracellular ROS are one of the most damaging effects of UVB irradiation on the skin, with subsequent damage to cellular components directly, leading to cell death^25^. To examine the potential protective effect of DeinoPol on intracellular ROS generated by UVB irradiation, cells were exposed to UVB (120 mJ/cm^2^) in the presence of DeinoPol (10 μg), and intracellular ROS level was measured using DCFDA, a probe that becomes fluorescent when oxidized by free radicals. Intracellular fluorescence signal was high in NHEK-Ad cells that did not receive DeinoPol treatment (Fig. 5C). However, ROS content was significantly attenuated in the DeinoPol pre-treated group, similar to that in non-irradiated cells. These results indicate that DeinoPol has potential protective effect for a human keratinocyte cell line (HaCaT) against radiation-induced oxidative stress.

Another indicator of ROS-induced cell damage is wound repair, since cells irradiated with UV or treated with H_2_O_2_ may be inhibited in migration and proliferation^25,26^. To determine whether pre-treatment with DeinoPol protects cells from damage caused by ROS and further enhances cell proliferation, we performed a wound-healing assay (Fig. 5D). Because of the slow growth of NHEK-Ad, we used keratinocyte cancer cell line (HaCaT) for this experiment. Under normal conditions, the scratched wound of the HaCaT monolayer was completely closed within 18 h, but when cells were treated with 1000 μM of H_2_O_2_, the wound was not completely closed until after 18 h, indicating that ROS-induced cell damage caused the reduction of wound healing. However, when cells were treated with H_2_O_2_ in the presence of DeinoPol, the wound area of the cell monolayer closed more than it did in the cells that did not receive DeinoPol pre-treatment. These results indicate that DeinoPol protected cells from ROS-induced damage and led to more efficient cell migration and proliferation.

### Inhibition of apoptosis-related signaling pathway by treatment with DeinoPol

UV-induced ROS can lead the depolarization of the mitochondrial membrane, stimulating Bcl2/Bax pro-apoptotic protein expression and activating procaspase-3 to cause cell death via apotosis^27–29^. To examine the effect of DeinoPol on UV-induced apoptosis, NHEK-Ad cells were irradiated in the presence of DeinoPol (10 μg), and Bax2 and Bax protein expression was then determined (Fig. 5E). Bcl2 protein expression in keratinocytes was significantly lower and Bax protein expression was concomitantly higher after 120 mJ/cm^2^ UVB irradiation compared to those in non-irradiated control. In contrast, irradiated cells in the presence of DeinoPol (10 μg) showed reduced Bax expression and increased Bcl2 expression, similar to those in non-irradiated cells. A previous study showed that increasing the Bax/Bcl-2 ratio up-regulates caspase-3 to promote cell apoptosis^29^. We next examined caspase 3/7 activity in NHEK-Ad cells under irradiation (Fig. 5F). Without irradiation, the activity of caspase 3/7 was barely detected in NHEK-Ad cells. When cells were irradiated with 120 mJ/cm^2^ of UV, caspase3/7 activity dramatically increased, reaching 45 kU, but its activity significantly decreased to ~32 kU when pre-treated with DeinoPol, indicating that DeinoPol can inhibit cell apoptosis via caspase-mediated pathways.

## Discussion

UV radiation induces water radiolysis and produces hydroxyl radicals by the Fenton or Haber–Weiss reaction^30,31^. This oxidative stress causes various cutaneous lesions, such as photoaging and photocarcinogenesis^1^. In addition, chronic exposure to UV radiation damages the integrity of the extracellular matrix in the skin tissues, and this damage is responsible for skin wrinkling, laxity, dryness, and thickness^2,3^. *Deinococcus* spp. have highly diverse and redundant antioxidant defense systems^14,15,32^. The ROS-scavenging system is composed of several lines of enzymatic and non-enzymatic antioxidants such as catalase, peroxidase, superoxide dismutase, lipoic acid, intracellular manganese, pyrroloquinoline quinone (PQQ), and carotenoids^5,9,17,33^. Bacterial EPSs are involved in various biological functions such as storage of energy, cell wall architecture, cellular communication, molecular organization, and stress resistance^9^. To our knowledge, this is the first study to show that deinococcal EPS, also called DeinoPol, is another type of antioxidant involved in resistance to severe environmental stresses, including UV radiation and hydroxyl radicals.

To date, most industrially important EPSs are produced in plants, animals, or algae, because of the low cost of downstream processing^10,34,35^. However, the use of plant- and animal-derived resources is becoming limited because of environmental disruption, depletion, and contamination. As an alternative, several bacterial EPSs have been reported during recent decades as a source of high-value biomaterials to produce cosmetics, pharmaceuticals, and natural medicines^11,36^. Dextran, discovered in the mid-19^th^ century, was originally used in solution as a plasma expander^9^. Researchers are now focused on isolating structurally and functionally identical EPSs from bacteria, such as those isolated from extreme environments (deep seas, hot springs, Antarctic areas). These organisms are attractive sources for valuable and identical EPSs; *D. radiodurans* is one such species that produces several valuable molecules, but the structure and function of these molecules have not been reported yet.

Because of the antioxidant effects of DeinoPol, its application in cosmetics and biomedicine is an attractive research area. However, to create a high-value medical polymer, it is crucial to reduce production costs and obtain a high degree of purity without loss of the original functional properties^20,37^. To improve the productivity of DeinoPol, it is crucial to optimize the fermentation process. Bacterial EPS productivity is highly influenced by media components and cultivation conditions, such as carbon source, temperature, and growth phase. Production of GalactoPol and FucoPol isolated from *Pseudomonas oleovorans* and *Enterobacter* A47, respectively, are maximized under aerobic conditions, with carbon availability concomitant with nitrogen and oxygen limitation^38^. In contrast, gellan and cellulose production is favored in media supplemented with vitamins and amino acids^39–44^. We also investigated the optimal culture condition for DeinoPol production based on its sugar composition analyzed by Bio-LC mass spectrometry in this study. We examined fucose, galactose, rhamnose, glucose, arabinose, mannose, xylose, and fructose as carbon sources but did not find significant enhancement of DeinoPol production in the media supplemented with different carbon sources (Supplementary Fig. 03). Another strategy to improve EPSs in bacteria is genetic modification of the EPS biosynthesis pathway. When the *dra0033* gene encoding ExoP-like protein was deleted, DeinoPol production was significantly reduced. We observed that overexpression of both *dr0054* and *dr0055* genes increased DeinoPol production by approximately 60% (Korea Patent:10-2018-0030335). Further biosynthesis mechanisms of DeinoPol, as well as the influence of DR0054 and DR0055 proteins on DeinoPol production, should be investigated to determine how DeinoPol can be produced in the cosmetic industry.

In addition to enhancing productivity, the required degree of DeinoPol purity is another criterion for its application in biomedical and cosmetic industries. Most bacterial EPSs are purified by cell lysis and precipitation with water-miscible solvents (e.g., methanol, ethanol, or isopropanol)^19,39,40^. We used gradient ethanol precipitation to purify DeinoPol, but its high toxicity (LD_50_ = 12.5 μg/mL) presents a limitation for its use as a cosmetic ingredient. We, therefore, used the Sevage method after ethanol precipitation to remove toxic, insoluble materials such as lipids and membrane proteins^41–43^. The final product was shown to be non-toxic at over 1000 μg/mL in NHEK-Ad (Supplement ary Fig. 2). However, the purity of DeinoPol was likely to be less than 90% based on gel filtration chromatography and on protein and nucleic acid concentration analyses. To obtain a higher grade of DeinoPol, additional processes should be performed following precipitation and the Sevage method, such as protein removal by protein precipitation with trichloroacetic acid or proteinase treatment^42,43^.

Research interest in bacterial EPSs continues to increase and is focused on isolating new polymers with identical health-associated effects^8,45^. To our knowledge, we are the first to isolate and characterize a deinococcal EPS and report its possible application as an anti-ROS agent. Next, we will further develop the production and purification process to reduce manufacturing costs and enhance purity of the final product, anticipating that new market needs could be created for value-added products in which traditional EPSs are unable to compete.

## Materials and Methods

### Mouse experiments

All animal experiments were conducted with the approval of the Committee on The Use and Care of Animals at Korea Atomic Energy Research Institute (KAERI-IACUC-2018-010) and performed according to accepted veterinary standards. Seven-week old female C57BL/6 mice were purchased from Orient Bio (Seongnam, Korea). Mice were intraperitoneally injected with *D. radiodurans* R1 or its isogenic mutant strain (10^8^ CFU/mice) in PBS. At 3 h post administration, mice were γ-irradiated at 13 Gy using Gammacell^®^40 Exactor (Best Theratronics Ltd; Ottawa, Canada) at Advanced Radiation Technology Institute, Korea Atomic Energy Research Institute (Jeoneupsi, Korea). Mouse blood, spleen, lungs, and liver were collected 3 days after administration. Tissues were homogenized by plunging in 1.5 ml of PBS through a 40 μm mesh strainer, and bacterial numbers were counted. Mice survival and body weight were monitored and recorded daily for 17 days.

### Bacteria strains and culture conditions

*Deinococcus radiodurans* R1 (ATCC13939) was obtained from the American Type Culture Collection (ATCC) and cultivated at 30 °C in TY broth containing 0.5% tryptone and 0.3% yeast extract or TGY broth containing 0.5% tryptone, 0.3% yeast extract, and 0.1% glucose^31^. All culture media used in this study was purchased from Difco (Franklin Lakes, NJ, USA).

### DRA0033 mutant construction

The DRA0033 mutant strain was constructed using the double cross-over recombination method as previously described^46^. The 1024 bp upstream and 718 bp downstream of *dra0033* were amplified with the primer upstream set (3803:AAG GTA CCG CCC CAA ACA GTT TC and 5803:TTC TCG AGA ACG CTC CAG TTC GG) and downstream set (3804:AAG GAT CCG CCG CGC TCA GGG CTC and 5804:TTG CAT GCA GCG CGG GGT TAT C) and cloned into the pKatCAT plasmid. The cloned plasmid was transferred into *D. radiodurans* R1 as previously described^46^, and chloramphenicol-resistant transformants were selected on TGY agar plates. Gene replacement was confirmed by diagnostic PCR using primers (3805:TGT GGG TCT GGA CAC GGG CG and 4805:AGG AAC AAA CCA ACA ACA GA) that bind outside the mutant cassette on the genomic DNA of *D. radiodurans* R1.

### DeinoPol purification

DeinoPol was extracted, isolated, and purified as described in previous studies^39–42^ with slight modifications. *D. radiodurans* R1 strain was cultured in 4 L of TGY broth at 30 °C, shaking at 900 rpm. After 48 h of incubation, the culture was mixed with 0.1% deoxycholate to lyse the bacterial cell wall and heated at 100 °C for 10 min to inactivate the bacteria and enzymes. Then, the cells were removed by centrifugation at 10,000 × *g* for 30 min at 4 °C. The supernatant was concentrated and dialyzed using a Minimate tangential flow filtration system with 30 K Minimate capsule (Pall Life Sciences; Port Washington, NY, USA). The concentrated supernatant (approximately 40 mL) was precipitated with 160 mL of 95% ethanol (Daejungchem; Seoul, Korea) at 4 °C for 12 h, and the precipitate was collected by centrifugation at 5000 × *g* for 10 min at 4 °C to yield the crude polysaccharide solution. The proteins in the crude polysaccharide were removed by the Sevage method as described previously^41,42^. Briefly, the precipitate was dissolved in 10 mL of distilled water and mixed with 30 mL chloroform:*n*-butanol (4:1 v/v). After shaking vigorously for 5 min, the mixture was allowed to stand for 15 min, and the aqueous phase was collected and precipitated with 80% ethanol. After filtration with a 0.22 μm Millex-GP syringe filter unit (Merck Millipore; Burlington, MA, USA), the final product was lyophilized.

### DeinoPol quantification

Total carbohydrates were estimated using the anthrone method as described previously^21^. Lyophilized DeinoPol was dissolved in 0.2 mL of distilled water and mixed with 0.8 mL of 0.2% anthrone solution in sulfuric acid. DeinoPol was hydrolyzed into simple sugars at 90 °C for 10 min and cooled to room temperature. The optical density was then measured at 620 nm using a Victor X3 light plate reader (PerkinElmer, Waltham, MA, USA). d-glucose was used as a standard, and values are represented as percent of dry weight of the samples. The concentration of DNA and protein in purified DeinoPol was measured using the NanoVuePlus spectrophotometer (GE Healthcare; IL, USA).

### Carbohydrate composition analysis

Monosaccharide analysis was performed using a CarboPac^TM^ PA-10 column (guard column 4 × 50 mm) (Dionex, Sunnyvale, CA, USA) coupled to a Dionex DX500 series chromatograph (Dionex) at the Korea Basic Science Institute (Ochang, South Korea), as described previously^47^. In brief, DeinoPol (10 mg) was dissolved in 0.5 M trifluoroacetic acid (5 mL) and incubated for 24 h at 60 °C followed by purification using Bio-Gel P-4 (200–400 mesh) (Bio-Rad, Hercules, CA, USA). The hydrolyzed monosaccharides were analyzed with Bio-LC high-performance chromatography. Data were processed using Thermo Scientific^TM^ Chromeleon^TM^ Data System software (Waltham, MA, USA).

### Biofilm quantitative assay

Biofilm quantification was performed by crystal violet staining, as described previously^48^. Overnight cultures of *D. radiodurans* R1 and its isogenic mutant (Δ*dra0033*) were diluted 1:100 in 2 × TGY broth and transferred into flat-bottomed polystyrene 96-well micro-titer plates (SPL, Pocheon, South Korea). After 48 h incubation at 30 °C, unattached bacteria were gently washed with 300 μL phosphate buffer saline (PBS; Lonza, Basel, Switzerland) and stained with 0.2% crystal violet solution for 1 min. Thereafter, excess crystal violet was removed by washing five times with 300 μL PBS. Crystal violet was solubilized with 200 μL acetone:ethanol (2:8 v/v). Supernatants were transferred onto a new flat-bottomed 96-well plate, and the optical density was measured at 570 nm using a Victor X3 light plate reader (PerkinElmer, Waltham, MA, USA).

### Scanning electron microscopy (SEM) analysis

*Deinococcus radiodurans* R1 and its isogenic mutant (Δ*dra0033*) were harvested at A600 = 0.5 and fixed overnight at 4 °C in 4% glutaraldehyde. After centrifugation, the pellet was washed three times with PBS and dehydrated through a graded ethanol series (35%, 50%, and 70%), followed by drying the cells in a drying chamber. The samples were gold-coated using a gold sputtering unit and observed using a JEOL JSM-840 scanning electron microscope (Tokyo, Japan) at the Seoul National University.

### Deinococcal survival assay

*Deinococcus radiodurans* R1 and its isogenic mutant (Δ*dra0033*) were cultured at log phase (A600 = 1.0) and the number of cells was adjusted to approximately 10^7^ CFU/mL in TGY broth. To test the resistance to environmental stress, cells were treated with hydrogen peroxide (Sigma-Aldrich; St.Louis, MO, USA) for 60 min or irradiated with UV light (302 nm) from the CL-1000 irradiator (UVP; Upland, CA, USA) or γ-irradiated for 60 min using a ^60^Co-gamma irradiator (point source, AECL, IR-79; MDS Nordion International Co., Ltd.; Ontario, Canada) at the Advanced Radiation Technology Institute, Korea Atomic Energy Research Institute, with an absorbed dose of 5–15 kGy. For survival under desiccation, 10 μL of bacteria-containing medium was placed on the glass slide (SPL) and then stored in a desiccation jar for 3 days, followed by washing the cells with 100 μL PBS and spotting onto TGY agar plates serially.

### Cell culture conditions

Primary human epidermal keratinocytes (NHEK-Ad) and an immortal human keratinocyte cell line (HaCaT) were obtained from Lonza and the Korean Cell Line Bank (Seoul, Korea). The cells were cultured in KBM^TM^-2 medium (Lonza; Basel, Switzerland) with KGM^TM^-2 growth supplement (Lonza), 100 U/mL penicillin and 100 μg/mL streptomycin (Gibco; Grand Island, NY, USA) at 37 °C in a humidified chamber supplemented with 5% CO_2_.

### Cell proliferation assay

A Cell Counting Kit-8 (CCK-8: Dojindo; Kumamoto, Japan) was used to measure the cytotoxicity and proliferation of NHEK-Ad. Cells were plated at a density of 10^4^ cells per well in a 96-well cell culture plate (SPL); 5 μL CCK-8 was added to each well and incubated for an additional 2 h at 37 °C. Viable cells were estimated by measuring the optical density at 450 nm using a Victor X3 light plate reader (Perkin-Elmer).

### Measurement of intracellular ROS

Human adult primary keratinocytes (NHEK-Ad, Lonza) were plated on black 96-well cell culture plates (SPL). When cells reached 70–80% confluence, 20 μL DCFDA/H_2_DCFDA (Abcam; Cambridge, UK) was pre-loaded to each well followed by UV exposure. The fluorescence intensity of each well was measured 30 min after irradiation with excitation 485 nm and emission 530 nm, using a Victor X3 light plate reader (Perkin-Elmer) or observed immediately under a fluorescence microscope (Olympus; Tokyo, Japan).

### *In vitro* wound healing assay

Human keratinocyte cell line (HaCaT) was seeded in a tissue culture 6-well plate at an initial density of 3 × 10^4^ cells/well overnight. When cells reached 95% confluence, a micropipette tip was used to scratch a wound on the monolayer as described previously^26^. Wells were then treated with H_2_O_2_ and/or DeinoPol (10 μg), and wound closure was observed by phase contrast microscopy (Nikon; Tokyo, Japan). Digital images were taken at 18 h.

### Western blotting analysis

NHEK-Ad cells (10^5^) were plated on 6-well cell-culture plates (SPL). At 80% confluence, the wells were pre-treated with DeinoPol (10 μg) followed by irradiation with 120 mJ/cm^2^ UV light. After 6 h of incubation at 37 °C, cells were lysed with RIPA buffer (Sigma-Aldrich) and separated on Bis-Tris Bolt gel (Invitrogen; Carlsbad, CA, USA) followed by transfer onto nitrocellulose membranes (Bio-Rad; Hercules, CA, USA). Membranes were blocked in 5% skimmed dry milk (Bio-Rad) in 0.05% Tween-20 in PBS (PBS-T) and then incubated with anti-Bcl-2 IgG1 monoclonal antibody (Santa Cruz Biotechnology; Santa Cruz, CA) or anti-Bax IgG2b monoclonal antibody (Santa Cruz Biotechnology). The membrane was then washed and incubated with horseradish peroxidase (HRP)-conjugated rabbit anti-mouse immunoglobulin (Southern Biotech; Birmingham, AL, USA). Membrane-bound peroxidase was detected by TMB-ELISA substrate solution (Thermo-Fisher; Waltham, MA, USA).

### Caspase 3/7 activity assay

Caspase 3/7 activity was analyzed using a Caspase-3/7 fluorescence assay kit (Cayman Chemical; Ann Arbor, MI, USA) following the manufacturer’s protocols. In brief, 10^4^ NHEK-Ad cells were plated into 96-well cell culture plates (SPL). At 80% confluence, the cells were irradiated with 120 mJ/cm^2^ UV light with or without pre-treatment of DeinoPol (10 μg). After 3 h, cells were lysed, followed by incubation of 90 μL cell lysate and 10 μL caspase 3/7 substrate solution. The fluorescence intensity of each well was measured with excitation 485 nm and emission 535 nm using a Victor X3 light plate reader (PerkinElmer).

### Cell apoptosis analysis

NHEK-Ad cells (10^4^) were plated on 6-well cell culture plates (SPL). At 80% confluence, the cells were irradiated with 10 Gy of γ-radiation using a Gammacell^®^ 40 Exactor (Best Theratronics; Ottawa, Canada) at the Advanced Radiation Technology Institute, Korea Atomic Energy Research Institute. Cells were trypsinized, washed, and stained with Dead Cell Apoptosis Kit with Annexin V-Alexa Fluor 488 and PI (BD Bioscience; San Jose, CA, USA) and incubated for 15 min at 37 °C in the dark. Cells were analyzed by MACSQuant flow cytometry (Miltenyi Biotech; Bergisch Gladbach, Germany). TUNEL assay was also used to determine the effect of DeinoPol on radiation-induced cell apoptosis according to the manufacturer’s instructions (Promega). In brief, cells were treated with cold 4% paraformaldehyde for 30 min and 0.3% Triton X-100 was added for 5 min. The TUNEL detecting solution (terminal deoxynucleotidyl transferase and fluorescence solution) was added and allowed to stand for 60 min at 37 °C. Images were acquired using an Olympus IX3 inverted fluorescence microscope (Tokyo, Japan).

## Supplementary information

Supplementary Information

## Data Availability

The data sets generated and/or analyzed during the current study are available from the corresponding author on reasonable request.
