# Supplementary material for: Antioxidant Activities of an Exopolysaccharide (DeinoPol) Produced by the Extreme Radiation-Resistant Bacterium Deinococcus radiodurans
Source: Sci Rep. 2020 Jan 9;10:55. doi: 10.1038/s41598-019-56141-3 (PMC6952347; doi:10.1038/s41598-019-56141-3)
Supplement: Supplementary file 1 — Supplementary Information [file 41598_2019_56141_MOESM1_ESM.pdf]

Antioxidant Activities of an Exopolysaccharide (DeinoPol) Produced by the Extreme Radiation-Resistant Bacterium *Deinococcus radiodurans*

Shun Mei Lin,[1,2*] Chan Yu Baek,[1,3*] Jong-Hyun Jung,[1,4] Woo Sik Kim,[1] Ha-Yeon Song,[1] Ji Hee Lee,[1] Hyun Jung Ji,[1] Yong Zhi,[1,4] Bo Sun Kang,[5] Yong-Sun Bahn,[3] Ho Seong Seo,[1,4#] and Sangyong Lim[1,4#]

[1]Research Division for Radiation Science, Korea Atomic Energy Research Institute, Jeongeup, Republic of Korea
[2]R&D Center, MediNResearch, Jeongeup, Republic of Korea
[3]Department of Biotechnology, College of Life Science and Biotechnology, Yonsei University, Seoul, Republic of Korea
[4]Department of Radiation Biotechnology and Applied Radioisotope Science, University of Science and Technology, Daejeon, Republic of Korea
[5]Department of Radiological Science, Konyang University, Daejeon, Republic of Korea

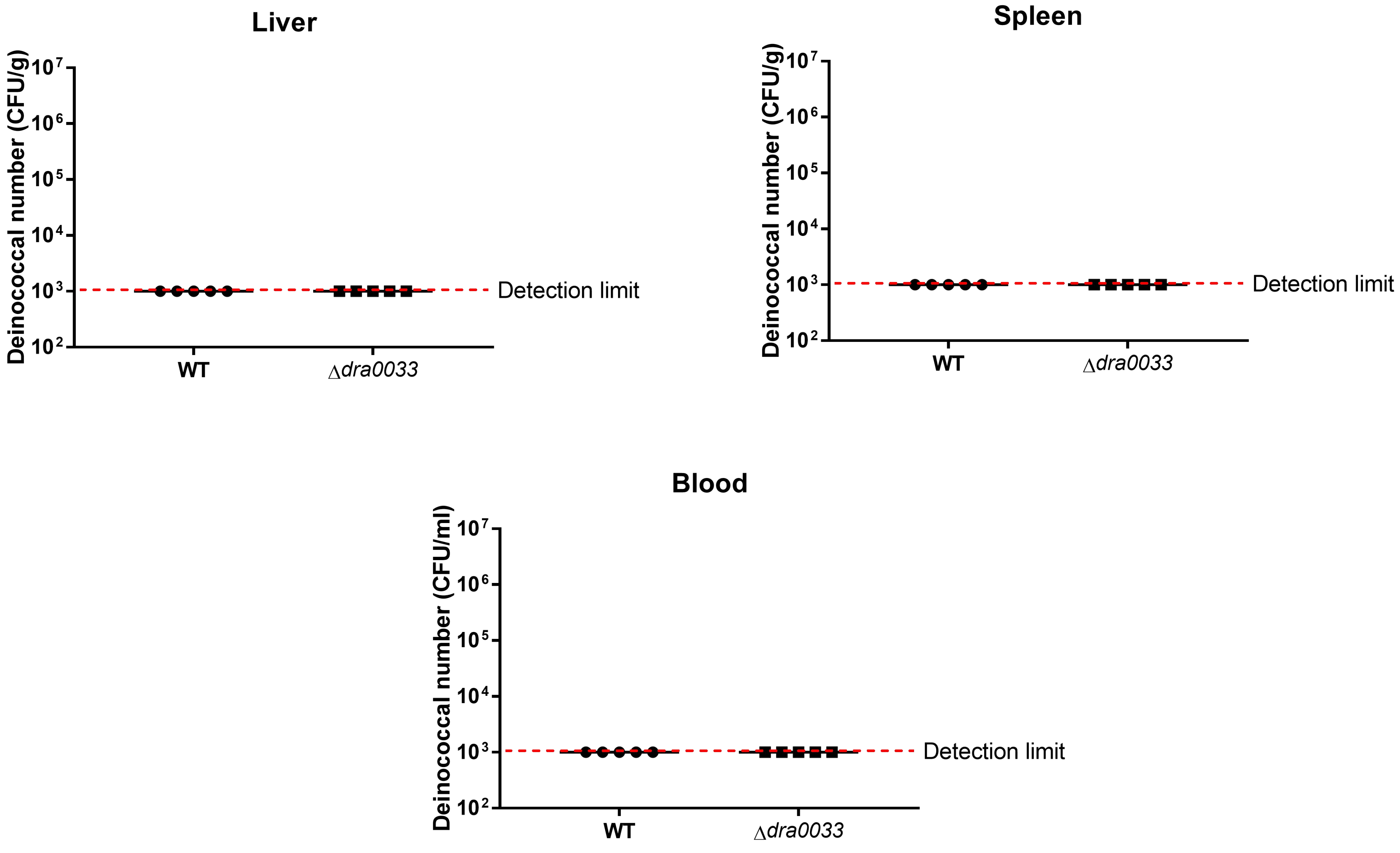

Supplementary Figure 01. Clearance of *D. radiodurans* in mice. (A-C) Mice (n=5 per group) were inoculated intraperitoneally (i.p.) with either wild-type R1 (WT) or Δ*dra0033* (10^8 CFU). Blood, liver, and spleen of mice were collected at 72 h after inoculation and plated onto TGY agar plates to measure the survival of *D. radiodurans* in mice. Detection limit indicates the minimum number of enumerated bacteria on a TGY agar plate (10^3 CFU).

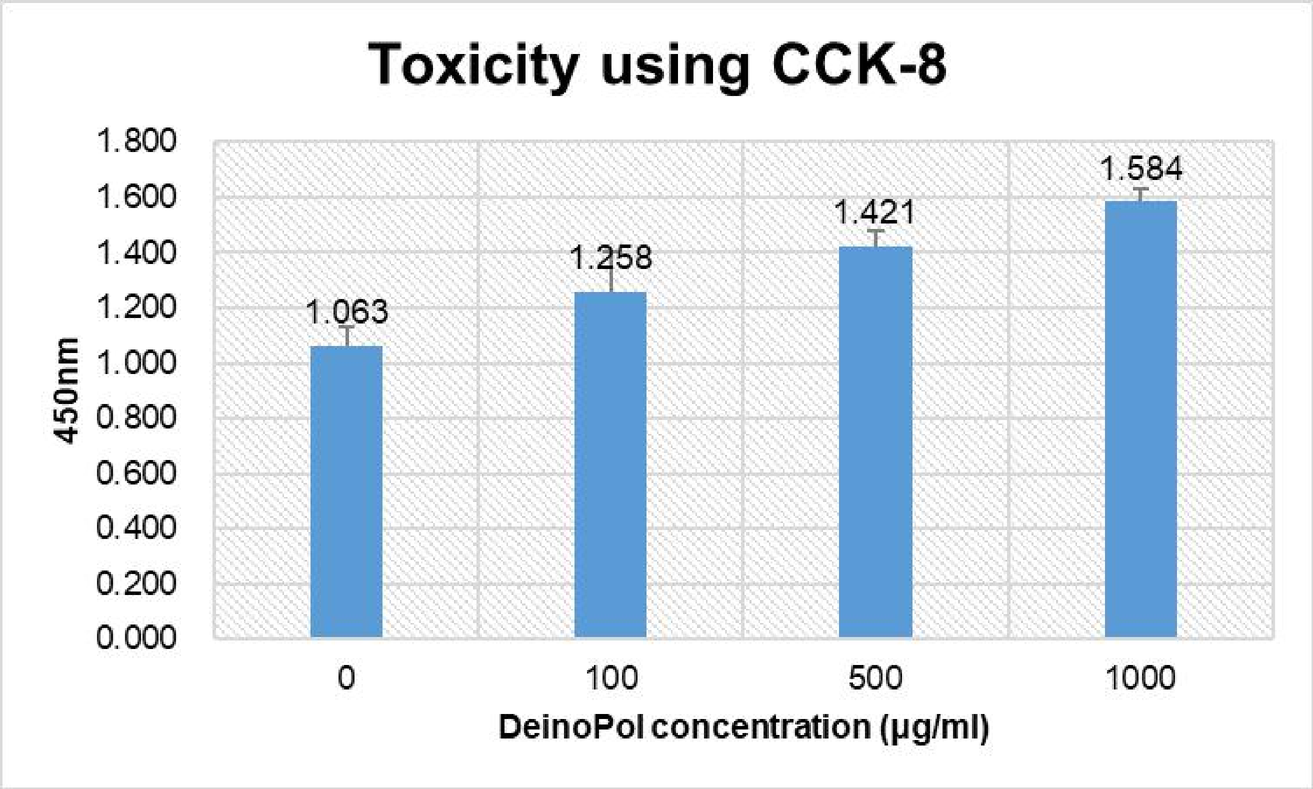

Supplementary Figure 02. Toxicity of DeinoPol. An approximately 70% confluent NHEK-Ad monolayer was incubated with the indicated c oncentration of DeinoPol for 24 h. Cell proliferation was measured by adding 5 μL CCK-8 and incubating for an additional 2 h at 37 °C. Viable cell number was estimated by measuring the optical density at 450 nm.

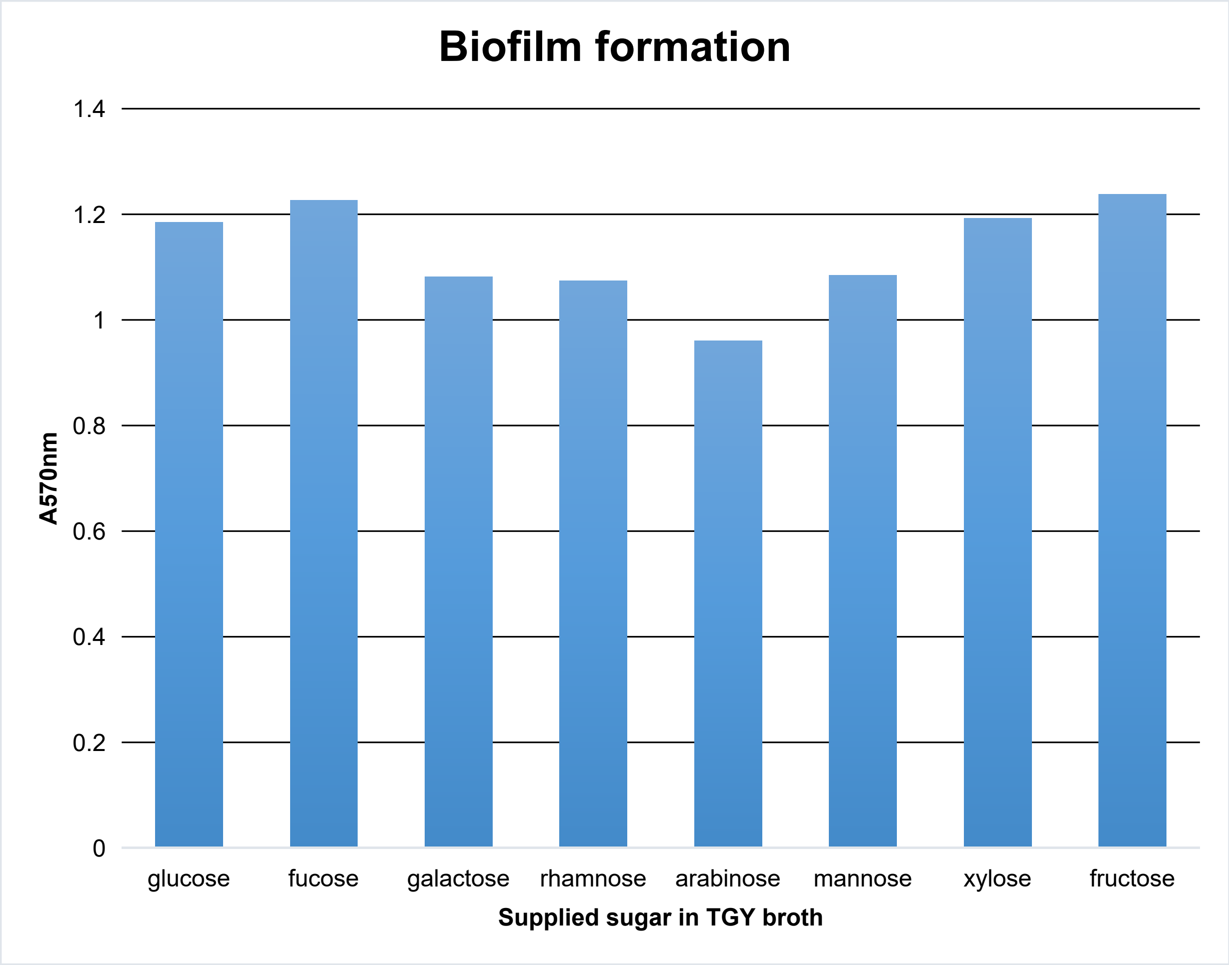

Supplementary Figure 03. Deinococcal biofilm formation in TGY broth with different sugar sources. *D. radiodurans* were seeded on a 96-well plate and incubated for 48 h at 30 °C. Levels of biofilm formation were measured by staining with 1% crystal violet, and absorba nce was read at 450nm.
